# Development and Validation of a Questionnaire on Motivation for Cooperative Playful Learning Strategies

**DOI:** 10.3390/ijerph18030960

**Published:** 2021-01-22

**Authors:** Ana Manzano-León, Pablo Camacho-Lazarraga, Miguel A. Guerrero-Puerta, Laura Guerrero-Puerta, Antonio Alias, José M. Aguilar-Parra, Rubén Trigueros

**Affiliations:** 1 Hum-878 Research Team, Health Research Centre, Department of Psychology, University of Almería, 04120 Almería, Spain; aml570@ual.es; 2 Centro Universitario San Isidoro, 41092 Seville, Spain; pcamacho@centrosanisidoro.es; 3 Department of Education, University of Seville, 41004 Seville, Spain; migupu97@gmail.com; 4 Department of Education and Social Psychology, Pablo Olavide University, 41013 Seville, Spain; laura.guerrero.puerta@gmail.com; 5 Department of Education, University of Almería, 04120 Almería, Spain; aag344@ual.es

**Keywords:** gamification, game-based learning, learning motivation, university education, cooperative learning

## Abstract

Playful learning strategies, such as educational gamification, game-based learning, and escape rooms are increasingly being incorporated into the university education system. In this study, it aims to develop and validate an instrument to analyze motivation regarding the use of playful learning strategies in university students. A total of 450 university students aged between 18 and 55 (Mean = 22.72; Standard Deviation = 5.01) were part of the sample, with whom playful strategies were implemented during the 2019/2020 school year. The results obtained in the confirmatory factor analysis indicate that the questionnaire on motivation for playful learning strategies has adequate psychometric properties to assess the motivation and perception of student learning in the implementation of ludic strategies in the classroom in the Spanish university context.

## 1. Introduction

During the last few decades, one of the concerns of university education has been referred to moving from a traditional approach to an educational paradigm focused on student activity [1]. Numerous authors [2,3] have proclaimed the need to create a more active and meaningful environment for university students, creating a paradigm shift in favor of a curriculum that responds to the challenges of today’s society, where the competence of learning to learn is favored. In this sense, new teaching strategies have emerged in recent years with the aim of transforming teacher–student transmissive education and adapting pedagogical practices to promote meaningful and committed learning experiences in preparing students for the world in which we live.

One of the educational strategies that has been gaining strength in the last 10 years is educational gamification [4] and other playful strategies that involve games, such as game-based learning [5], escape rooms [6], or serious games [7]. The common element of these learning strategies is the use of games or game elements to promote student motivation and work on curricular content and social and personal skills through aesthetics and game dynamics (in the case of gamification, escape rooms, breakouts) or games/video games (in the case of game-based learning and educational video games). These strategies have a direct relationship with playful learning. Playful learning is a pedagogical methodology where it is sought that children are active, engaged, socially competent, and can have materials that are fun and meaningful to them [8]. It can be highlighted from playful learning by providing an environment where students have the freedom to fail, where mistakes have no serious consequences in the real world. Playful learning follows the constructivism approach, seeking that students be active participants in their learning process, and tries to offer them surmountable challenges to learn while being intrinsically motivated [9].

From this perspective, these strategies are going to be deeply installed in different contexts, including education, due to the change of pace of the digital society [10]. On the other hand, games can be a facilitator to activate the commitment to the task, this having a direct relationship with academic motivation [11]. Academic motivation is defined as that which drives, leads, maintains effort, activates cognitive resources to learn [12], is dynamic [13], and has intrinsic and extrinsic reinforcers [14].

Intrinsic motivation more effectively increases engagement and performance than extrinsic motivation [15]. When students enjoy the game mechanics, learning is connected to a pleasant situation, enhancing intrinsic motivation. Intrinsic motivation is associated with flow, the mental state where an individual experiences high levels of concentration, enjoyment of energy, and engagement in an activity, where consequently, the game would be a reinforcer of successful motivation [16].

It is also highlighted that despite the fact that gamification and game-based learning can be perceived or analyzed as overly competitive strategies [17], they are an opportunity to develop collaboration between students, since if dynamics and mechanics are used cooperatives, teamwork can be promoted. An example is the study by Knautz, Wintermeyer, Orszullok, and Soubusta [18], where they created the collaborative gamified program “The Legend of Zyren” with game mechanics, such as points, leaderboards and levels, and a narrative. Their results showed that the students perceived the platform as useful, motivating, and fun. Playful design had a positive impact on content mastery and student performance with a positive correlation between players’ XP and their final grades. Other studies reinforce this idea, indicating that gamification methods are successful in promoting collaboration and this collaboration can positively affect the results of the course [19]. In higher education, the results of the meta-analysis of Subhash and Cudney [20] clarify that there are various benefits of the use of educational gamification, specifically higher student participation, motivation, perceived learning, and academic achievement. It also identifies that in higher education points, medals, rankings, levels, and graphics are mainly used as game elements. In the case of escape rooms, they are strategies that foster a deeper understanding of didactic content through playful challenges. When escape rooms are implemented in the university context, students are interested in the curricular content, and it proved to be effective in promoting teamwork and collaboration to achieve a common goal [21].

Different studies have analyzed the use of playful learning strategies; however, numerous studies develop ad hoc surveys or use qualitative techniques [22]. Some studies have focused on developing instruments aimed at assessing the influence of educational gamification from different perspectives. One of the most recognized is the Gameful Experience Scale (GAMEX) [23], designed and validated with a sample of 129 with an average age of 26.15 years. This Likert scale contains 27 items, divided into six dimensions: dominance (0.84), creative thinking (0.88), enjoyment (0.96), activation (0.87), absorption (0.91), and absence of negative affect (0.85).

Regarding competitive playful strategies, Baydas and Cicek [24] created and validated a scale to measure the impact of the use of Kahoot! in the classroom, with a sample of 91 university students (65.93% men), and their ages ranged between 18 and 21. This scale includes 23 items based on a 5-point Likert type with verbal anchors of 5 (Strongly Agree), 4 (Agree), 3 (Neither Agree nor Disagree), 2 (Disagree), and 1 (Strongly Disagree) with six main themes: learning effect, expected outcome, competition, entertainment, engagement, and intention. Bartlett’s sphericity test was found significant as *p* < 0.01. 

On the other hand, although it is not a specific instrument for the educational context, it is also worth highlighting the Gamification User Types Hexad Scale, which assesses user preferences in games. The scale consists of 24 items distributed in six factors: philanthropic, socializing, free spirit, winner, disruptor, and player. The authors used a sample of 556 adults. The Bartlett sphericity test was significant for both samples: (χ2 (276) = 1782.1, *p* < 0.001 for the English sample, and; χ2 (276) = 3771.9, *p* < 0.001 for the Spanish sample [25].

Högberg, Hamari, and Wästlund [26] have presented the Gameful Experience Questionnaire (GAMEFULQUEST) that is a validated instrument for measuring gameful experiences when using a service. It was validated with a sample of 371 adults (M = 38). The model has seven dimensions (Accomplishment, Challenge, Competition, Guided, Immersion, Social Experience, Playfulness). The correlation matrix showed coefficients above 0.3 between most items, where their respective predicted dimension and Bartlett’s test of sphericity was significant (*p* < 0.001).

All these questionnaires analyze motivation and academic performance through playful strategies. Despite having scientific utility, we currently do not have instruments to measure university students’ perception of their own learning and motivation in cooperative learning. During this research, a questionnaire has been designed to assess the perception of motivation, learning, flow, and teamwork; it can be used regardless of the type of playful strategy. To do this, once the scale was drawn up, a Confirmatory Factor Analysis (CFA) was done to ensure content validity, internal consistency, and factorial structure.

## 2. Materials and Methods

### 2.1. Participants

The sample of this study was made up of 450 university students, aged between 18 and 55 (Mean = 22.72; Standard Deviation = 5.01), with 183 men (40.70%) and 277 women (59.30%), after the application of a gamification program in the practical groups throughout the four-month period, being selected through accidental non-probabilistic sampling. The criteria for participation in the study was to be a university student, participate in the educational gamification program, and deliver signed, informed consent.

### 2.2. Instrument

The Questionnaire on motivation for cooperative learning play strategies (CMELAC) was used. The instrument was developed for the present study, and in its preliminary version, the scale consisted of 22 Likert-type items with a range of 1 to 5 (1 = Totally disagree, 2 = Disagree, 3 = Neither agree nor disagree, 4 = Agree and 5 = Totally agree). To ensure the validity of the content, this first version was reviewed by a group of experts in Educational Psychology, Gamification, and Game-Based Learning, with whom various meetings were held in which the statement of the different items that made it up was discussed and reviewed.

### 2.3. Procedure

To carry out the data collection, in the first place, an educational gamification program was designed for different subjects of the degrees of Social Education, Primary Education, and Early Childhood Education with a duration of one semester, directed by experts in educational gamification. A Small Private Online Course (SPOC) program [27] was implemented with a gamification system. In this SPOC, the students were divided into small groups of three to six students, and had to carry out different challenges related to the content of the subject. These curricular challenges had rubrics, and depending on the quality of their projects, they could win medals of different colors. In addition, the entire subject was presented with a superhero aesthetic, where the educational materials maintained that aesthetic. If, as a large group, they achieved a minimum of medals, doing the challenges set out in the SPOC, they managed to unlock the virtual escape room. The virtual escape room consisted of defeating the villain and saving the city. To do this, five screens were designed with playful challenges (locks, puzzles, secret codes, etc.) and quizzes of the subject content.

The research team contacted teachers who taught subjects in these grades to request their participation in the study. These gamified activities were collected in the teaching guide of the curricular practices of three subjects, and the students were informed of the purposes of the research and of the confidentiality of the data, and their authorization to participate was requested, following the recommendations of the American Psychological Association. The informants’ consent to participate in the research was obtained in writing before the questionnaire was applied. Previously, all the information related to the research and use of the data obtained for their publication was explained to the students, and enough time was offered to review all the information, as well as ask the necessary questions.

Before administering the scale to all students, a small group of people completed it to ensure that all items were understood correctly. The questionnaires were filled out in the classroom during school hours, individually and anonymously. The main researcher was present at the time the participants completed the questionnaires, noting that there were no wrong answers, that they could answer honestly, and that they could express any type of doubt during the process. The time to complete the questionnaire was approximately 10 min.

### 2.4. Data Analysis 

To determine the validity and reliability of the CMELAC, its psychometric properties were analyzed. A confirmatory factor analysis (CFA) was performed to test its factor structure. In addition, descriptive statistical analyses were performed and the reliability of the instrument was tested by internal consistency analysis (Cronbach’s alpha). Next, temporal stability (intraclass correlation, ICC) and multigroup analyses were performed to analyze gender invariance.

## 3. Results

### 3.1. Confirmatory Factor Analysis

**Figure 1:**
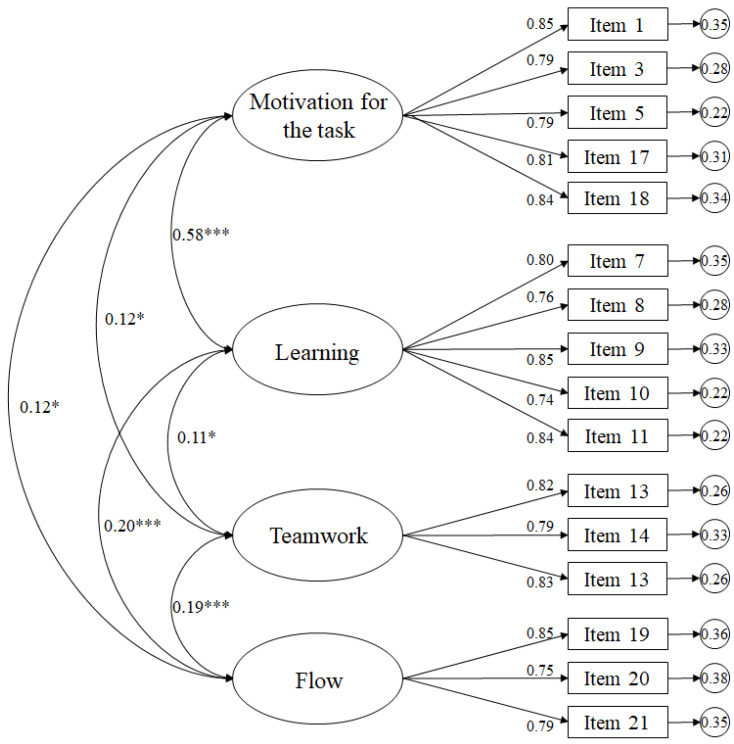
The ellipses represent the factors, and the rectangles represent the different items. The residual variances are shown in the small circles. *** *p* < 0.001; * *p* < 0.01

The 23-item eight-factor model was initially evaluated, with adjustment indices: χ2 (224, *N* = 450) = 2604.42, *p* = 0.001; χ2/df = 11.63; CFI = 0.70; TLI = 0.70; IFI = 0.70; RMSEA = 0.097 (90% CI = 0.082–0.110); SRMR = 0.086. However, we considered ostensible improvements in the factor structure of the questionnaire after analyzing the standardized residual covariance matrix, where we observed possible improvements, since the residual values of some elements correlated with the residual values of other elements and were associated with standardized residual >|2.00|. Thus, five items were removed from the model. Without these four items, the model’s fit indices were: χ2 (129, N = 450) = 671.82, *p* = 0.001; χ2/df = 5.21; CFI = 0.86; TLI = 0.86; IFI = 0.86; RMSEA = 0.077 (90% CI = 0.072–0.083); and SRMR = 0.071. Therefore, after observing these data, we proceeded to eliminate those items whose regression weights were less than 0.5, eliminating an item with two factors (motivation towards the task and teamwork). Excluding these two items, the model’s fit indices improved: χ2 (98, N = 450) = 338.50, *p* = 0.001; χ2/df = 3.45; CFI = 0.95; IFI = 0.95; TLI = 0.94; RMSEA = 0.061 (90% CI = 0.051–0.067); SRMR = 0.052. The final model (Figure 1) had standardized residual values (below two in absolute values) and the standardized regression weights were statistically significant (*p* < 0.001), ranging from 0.75 to 0.85.

### 3.2. Analysis of Invariance with Respect to Gender

Table 1 shows that the questionnaire is invariant with respect to gender.

### 3.3. Internal Consistency Analysis, Descriptive Statistics, and Bivariate Correlations

Table 2 shows the correlation analyses, positive between factors, the internal consistency analysis and the mean and standard deviation.

## 4. Discussion

The objective of this study was to elaborate and validate an instrument that allows assessment of the perception of students about their motivation, learning, teamwork, and flow acquired through playful learning strategies in educational contexts. Knowing the perception that students have about the development of playful activities in the classroom can offer very enriching information to explain the acquisition of curricular and social competences, as well as the engagement that can occur in students with the use of educational games.

In this sense, in the last decade we can see a growing interest in the implementation of gamification programs aimed at improving the acquisition of curricular content in both primary [28] and secondary [29] as a university education [30]. In the same way, it has also been studied how play can be a great tool to work on social skills [31]. However, despite the fact that more and more studies are interested in playful learning strategies, there are still not enough instruments that comprehensively assess these techniques in the educational context, especially for those programs that use cooperative learning.

Thus, this study offers the scientific and educational community a possibility to assess these aspects in the specific context of university education. Specifically, in this work, the Questionnaire on Motivation by Cooperative Learning Strategies (CMELAC) for university students and the different tests that support its factorial structure, validity, and internal consistency have been presented. The CFA revealed as the most appropriate model the one formed by 16 items and four factors (Motivation, Learning, Teamwork, and Flow). The questionnaire analyzes the main sensitive aspects of the use of cooperative playful strategies in the classroom. 

In the first place, this questionnaire assesses academic motivation in educational gamification systems, since it has been a main topic in subsequent studies (for example, [32]). The theory of gamified learning relates the influence of gamification with better attitudes and behaviors (motivation), which can, in turn, indirectly influence students’ learning outcomes [33]. Secondly, it measures the learning perceived by students after the gamification program. Gamification can help students to be clear about their objectives and consequently have more active participation in the classroom, and it can also satisfy their status recognition needs through game mechanics, and have them favor teachers’ feedback. This allows us to affirm that gamification can be a valuable educational strategy to improve student learning [34]. Third, this questionnaire seeks to be useful to evaluate cooperative gamified programs. For this reason, a key factor is teamwork. Teamwork in education consists of working with cooperative methodologies where a group of students work to achieve a shared goal. Educational gamification is a flexible educational strategy where it is possible to choose what type of activities (individual or cooperative) should be provided to students. When gamified cooperative activities are carried out, teamwork can improve, as well as motivation for the task [35]. Finally, flow is a state of total immersion, and a fusion of action and awareness associated with positive motivational experiences [36]. Gamification tries to use fun and meaningful activities to work on students’ skills and competencies. These activities should present a challenge for the students, managing to create an optimal flow circumstance for learning and behavior regulation [37].

Descriptive statistical analyses and reliability analyses show a positive correlation between the factors, in line with the results achieved in the confirmatory factor analysis. The reliability analysis of each of the factors reaches a Cronbach’s alpha score that is higher than 0.70, which shows that the distribution of the items is adequate [38]. 

Taking into account the different analyses carried out to verify the suitability of this instrument, the results support the validity of a structure made up of 16 items grouped into four first-order factors, also obtaining adequate internal consistency. For this reason, it can be affirmed that the CMELAC is shown as a valid and reliable instrument to assess the perception of students about their motivation, learning, teamwork, and personal skills acquired through playful learning strategies in educational contexts.

Despite the results obtained, some limitations of this study are discussed. First, the sample has not been probabilistic, and belongs to the same university. Second, factor analysis has shown evidence that the instrument can be used regardless of gender; however, future work should determine if it can also be used to establish differences according to other variables (e.g., age, socioeconomic status, college career).

The objective of this study has been to validate the Questionnaire on Motivation for Cooperative Learning Strategies (CMELAC; Appendix A and Appendix B) for the Spanish university population. The next step in our research will be to design and evaluate an educational gamification program with this instrument, in order to investigate whether the design of gamification can influence academic motivation and the acquisition of curricular and personal skills. In addition, it would be advisable to investigate the application of this questionnaire for the design of gamified learning environments, so that it can offer feedback to the teacher on their educational practices.

In conclusion, given the relevance of active learning strategies in the university context in recent years, and taking into account the specificity and reduced size of this scale, we believe that it could be used to identify and assess students’ perception of their motivation, learning, teamwork, and acquired personal skills. This would also make it possible to verify the effectiveness of the use of playful strategies in the educational context as a vehicle for the promotion of student motivation.

## Figures and Tables

**Table 1:** Multigroup analysis of invariance with respect to gender.

Models	*χ2*	*gl*	*χ2/gl*	Δ*χ2*	Δ*gl*	CFI	TLI	IFI	RMSEA (IC 90%)	SRMR
Model 1	610.31	196	3.11			0.94	0.94	0.94	0.055	0.043
Model 2	629.50	208	3.03	19.19	12	0.93	0.93	0.93	0.057	0.042
Model 3	658.46	218	3.02	48.15	22 **	0.93	0.92	0.93	0.057	0.042
Model 4	759.26	234	3.24	241.07	38 ***	0.92	0.92	0.92	0.061	0.046

** *p* < 0.01; *** *p* < 0.001.

**Table 2:** Analysis of reliability, mean, standard deviation, and bivariate correlations.

Factor	M	DT	α	1	2	3	4
1. Motivation for the task	4.12	0.96	0.78	-	0.54 ***	0.12	0.42 **
2. Learning	3.54	1.21	0.81		-	0.14 *	0.15 *
3. Teamwork	2.97	1.27	0.80			-	0.01
4. Flow	3.72	0.84	0.83				-

* *p* < 0.05; ** *p* < 0.01; *** *p*< 0.001. Note: M =Mean; DT= Typical deviation.

## Data Availability

Not applicable.

## Appendix A

**Table A1:** Cuestionario sobre la Motivación por las Estrategias Lúdicas de Aprendizaje Cooperativas (CMELAC).

This Scale Was Validated in SPANISH: Piensa en la Actividad/Actividades Lúdicas Realizada en Esta Asignatura y Contesta:
En general, he disfrutado de esta actividad lúdica
Repetiría este tipo de actividades
Me he sentido motivado/a
Mejoró mis conocimientos sobre la asignatura
Ha incrementado mi interés en la asignatura
Este formato de actividad ha sido apropiado para comprobar mis conocimientos de la asignatura
Me ayudó a identificar mis debilidades en la asignatura
Me ayudó a entender el contenido de la asignatura
Con este tipo de actividades aprendo más que en clases magistrales
Siento que pude relacionarme con mis compañeros/as de equipo para aprender
Aprendí de mis compañeros/as durante la actividad
Los elementos de juego me han parecido divertidos
Los elementos de juego me han motivado a la hora de realizar la actividad
Mientras jugaba no era consciente de lo que sucedía a mi alrededor
Me sentí capaz de realizar las actividades propuestas
Las actividades me parecieron reconfortantes y valiosas para mí

## Appendix B

**Table A2:** Questionnaire on Motivation for Cooperative Playful Learning Strategies (CMELAC) in English.

This Scale Was Validated in SPANISH: Think about the Playful Activity/Activities Carried out in This Subject and Answer:
In general, I have enjoyed this playful activity
I would repeat these types of activities
I have felt motivated
I improved my knowledge of the subject
My interest in the subject has increased
This activity format has been appropriate to check my knowledge of the subject
Helped me identify my weaknesses in the subject
It helped me understand the content of the subject
With these types of activities I learn more than in traditional classes
I feel like I was able to connect with my teammates to learn
I learned from my classmates during the activity
I found the game elements fun
The game elements have motivated me to carry out the activity
While playing I was not aware of what was happening around me
I felt capable of carrying out the proposed activities
I found the activities comforting and valuable to me

## References

[1] Vásquez B., Pleguezuelos C., Mora M.L. (2017). Debate como metodología activa: Una experiencia en educación superior. Univ. Y Soc..

[2] Kostiainen E., Pöysä-Tarhonen J. (2016). Meaningful Learning in Teacher Education, Characteristics of. Encyclopedia of Teacher Education.

[3] Nel L. (2017). Students as collaborators in creating meaningful learning experiences in technology-enhanced classrooms: An engaged scholarship approach. Br. J. Educ. Technol..

[4] Kirillov A.V., Vinichenko M.V., Melnichuk A.V., Melnichuk Y.A., Vinogradova M.V. (2016). Improvement in the Learning Environment through Gamification of the Educational Process. Int. Electron. J. Math. Educ..

[5] Sung H., Hwang G. (2013). A collaborative game-based learning approach to improving students’ learning performance in science courses. Comput. Educ..

[6] Kinio A., Dufresne L., Brandys T., Jetty P. (2019). Break out of the Classroom: The Use of Escape Rooms as an Alternative Teaching Strategy in Surgical Education. J. Surg. Educ..

[7] Arnab S., Lim T., Carvalho M., Bellotti F., de Freitas S., Louchart S. (2014). Mapping learning and game mechanics for serious games analysis. Br. J. Educ. Technol..

[8] Hassinger-Das B., Toub T.S., Zosh J.M., Michnick J., Golinkoff R., Hirsh-Pasek K. (2017). More than just fun: A place for games in playful learning. Infanc. Aprendiz..

[9] Whitton N. (2018). Playful Learning: Tools, Techniques, and Tactics. Res. Learn. Technol..

[10] Contreras R.S., Eguia J.L. (2016). Gamificación en Aulas Universitarias.

[11] Eseryel D., Law V., Ifenthaler D., Ge X., Miller R. (2014). An Investigation of the Interrelationships between Motivation, Engagement, and Complex Problem Solving in Game-based Learning. Educ. Technol. Soc..

[12] Valenzuela J. (2007). Más allá de la tarea: Pistas para una redefinición del concepto de motivación escolar. Educ. Pesqui..

[13] Otis N., Grouzet F., Pelletier L.G. (2005). Latent Motivational Change in an Academic Setting: A 3-Year Longitudinal Study. J. Educ. Psychol..

[14] Alawiyah T., Sulistiyo U. (2018). The Influence of Students Motivation Toward Students Achievement. Int. J. Lang. Teach. Educ..

[15] Deci E.L., Ryan R.M. (2012). Self-determination theory in health care and its relations to motivational interviewing: A few comments. Int. J. Behav. Nutr. Phys. Act..

[16] Wang L., Chen M. (2010). The effects of game strategy and preference–matching on flow experience and programming performance in game–based learning. Innov. Educ. Teach. Int..

[17] Furdu I., Tomozei C., Köse U. (2017). Pros and Cons Gamification and Gaming in Classroom. Brain.

[18] Knautz K., Wintermeyer A., Orszullok L., Soubusta S. (2014). From know that to know how—Providing new learning strategies for information literacy instruction. Commun. Comput. Inf. Sci..

[19] Yee N., Schroeder R., Axelsson A. (2006). The psychology of MMORPGs: Emotional investment, motivations, relationship formation, and problematic usage. Avatars at Work and Play: Collaboration and Interaction in Shared Virtual Environments.

[20] Subhash S., Cudney E.A. (2018). Gamified learning in higher education: A systematic review of the literature. Comput. Hum. Behav..

[21] Hermanns M., Deal B., Campbell A., Hillhouse S., Opella J., Faigle C., Campbell IV R. (2017). Using an “Escape Room” toolbox approach to enhance pharmacology education. J. Nurs. Educ. Pract..

[22] Aldemir T., Celik B., Kaplan G. (2018). A qualitative investigation of student perceptions of game elements in a gamified course. Comput. Hum. Behav..

[23] Eppmann R., Bekk M., Klein K. (2018). Gameful Experience in Gamification: Construction and Validation of a Gameful Experience Scale [GAMEX]. J. Interact. Mark..

[24] Baydas O., Cicek M. (2019). The examination of the gamification process in undergraduate education: A scale development study. Technol. Pedagog. Educ..

[25] Manzano-León A., Camacho-Lazarraga P., Guerrero-Puerta M., Guerrero-Puerta L., Alias A., Trigueros R., Aguilar-Parra J. (2020). Adaptation and Validation of the Scale of Types of Users in Gamification with the Spanish Adolescent Population. Int. J. Environ. Res. Public Health.

[26] Högberg J., Hamari J., Wästlund E. (2019). Gameful Experience Questionnaire (GAMEFULQUEST): An instrument for measuring the perceived gamefulness of system use. User Model. User Adapt. Interact..

[27] Ruiz-Palmero J., Fernández-Lacorte J.M., Sánchez-Rivas E. (2020). Colomo-Magaña, E. The implementation of Small Private Online Courses (SPOC) as a new approach to education. Int. J. Educ. Technol. High. Educ..

[28] López-Faican L., Jaen J. (2020). EmoFindAR: Evaluation of a mobile multiplayer augmented reality game for primary school children. Comput. Educ..

[29] Hashim H., Rafiqah M., Rafiq K., Yunus M. (2019). Improving ESL Learners’ Grammar with Gamified-Learning. Arab World Engl. J..

[30] Mora-Gonzalez J., Pérez-López I., Esteban-Cornejo I., Delgado-Fernández M. (2020). A Gamification-Based Intervention Program that Encourages Physical Activity Improves Cardiorespiratory Fitness of College Students: ‘The Matrix rEFvolution Program’. Int. J. Environ. Res. Public Health.

[31] Ouariachi T., Li C., Elving W. (2020). Gamification Approaches for Education and Engagement on Pro-Environmental Behaviors: Searching for Best Practices. Sustainability.

[32] Buckley P., Doyle E. (2014). Gamification and student motivation. Interact. Learn. Environ..

[33] Latifi G.R., Monfared M.P., Khojasteh H.A. (2020). Gamification and citizen motivation and vitality in smart cities: A qualitative meta-analysis study. GeoJournal.

[34] Bai S., Hew K.F., Huang B. (2020). Does gamification improve student learning outcome? Evidence from a meta-analysis and synthesis of qualitative data in educational contexts. Educ. Res. Rev..

[35] Chujitarom W., Piriyasurawong P. (2017). Animation Augmented Reality Book Model (AAR Book Model) To Enhance Teamwork. Int. Educ. Stud..

[36] Csikszentmihalyi M. (2008). Flow: The Psychology of Optimal Performance.

[37] Almqvist L., Uys C., Sandberg A. (2017). The concepts of participation, engagement and flow: A matter of creating optimal play experiences. S. Afr. J. Occup. Ther..

[38] Hambleton R.K., Muñiz J. (1996). Adaptación de tests para su uso en diferentes idiomas y culturas: Fuentes de error, posibles soluciones y directrices prácticas. Psicometría.

